# Extra-anatomical left common carotid and subclavian artery bypass followed by aortic arch replacement with frozen elephant trunk

**DOI:** 10.1186/s13019-023-02403-1

**Published:** 2023-10-09

**Authors:** Ryo Suzuki, Masafumi Akita, Suguru Miyazaki, Ryo Shimano

**Affiliations:** Department of Cardiovascular Surgery, Shinmatsudo Central General Hospital, 1-380 Shinmatsudo, Matsudo, Chiba 270-0034 Japan

**Keywords:** Frozen elephant trunk, Debranching-first technique, Total arch replacement

## Abstract

**Background:**

Total arch replacement (TAR) using a frozen elephant trunk (FET) allows for simultaneous treatment of the aortic arch and descending aortic pathology via median sternotomy. In addition, an extra-anatomical bypass performed between the left common carotid artery (CCA) and subclavian artery (SCA) prior to TAR allowed further proximalisation of the FET prosthesis, facilitated distal anastomosis of the TAR and spared the demanding left subclavian artery (LSA) anastomosis in the deep thorax. We investigated the efficacy of this debranching-first technique, followed by TAR using a frozen elephant trunk, as a two-stage operation for extensive thoracic aortic aneurysms in high-risk patients.

**Methods:**

Forty-nine consecutive patients with diffuse degenerative aneurysms from the aortic arch to the descending aorta or chronic aortic dissection who underwent left common carotid to subclavian artery bypass followed by TAR using a frozen elephant trunk and subsequent thoracic endovascular aortic repair between 2016 and 2021 were analysed. The baseline characteristics and clinical outcomes were assessed. The estimated overall survival, 5-year aortic event-free survival, and aortic reintervention rates were analysed.

**Results:**

The average European System for Cardiac Operative Risk Evaluation (*Euro*SCORE II) was 4.7 ± 2.5. The operative mortality rate was 4.1%, with no paraplegia events. The estimated 5-year overall survival, cumulative aortic-related mortality rates were 76.8% and 2%, respectively. The estimated 5-year overall cumulative aortic reintervention rate, including the intended intervention, was 31.3%. The estimated 5-year cumulative rate of non-intended reintervention was 4.5%.

**Conclusions:**

The assessed technique enables a less technically demanding surgery with reasonable outcomes. The estimated 5-year aortic event-free survival and reintervention rates were acceptable, suggesting that multiple stages of alternative open and endovascular interventions, such as this technique, may reduce the morbidity and mortality rates of high-risk patients with diffuse thoracic aortic aneurysm.

UMIN-CTR (University hospital Medical Information Network-Clinical Trial Registry)

https://center6.umin.ac.jp/cgi-open-bin/ctr_e/index.cgi

Clinical registration number: UMIN000051531

## Introduction

Diffuse aortic aneurysms involving the aortic arch to the descending aorta remain a great challenge for cardiovascular surgeons [1]. When performing median sternotomy and TAR, handling distal anastomosis remains a challenge if the pathology is located deep from the median sternotomy and if the aneurysm is large. However, the technical approach, which includes using a classical elephant trunk, makes a distal anastomosis proximal to the aneurysm and ostium of the LSA. Thus, it is easier to perform and less invasive than the conventional TAR [1]. This classical elephant trunk is required in a two-stage procedure; however, there is a significant interval mortality rate between the two procedures [2, 3]. Therefore, a less invasive one-stage operation to treat extensive aortic arches and descending aortic aneurysms has become popular. This allows the development of FET [4]. The FET technique, which involves a one-stage repair of extensive aortic arch aneurysms by combining the concepts of the classical elephant trunk principle and endovascular stenting of descending aorta pathology, was introduced in 2003 [5].

The debranching-first technique has been proposed by surgeons who aim for continuous blood flow to both hemispheres during the entire aortic arch procedure [6]. The advantage of this technique is that the LSA is already revascularised with supra-aortic bypass before TAR. The extra-anatomical bypass performed between the left CCA and SCA allows further proximalisation of the distal anastomosis of the aortic arch repair by covering the LSA orifice with a FET prosthesis. The method also helps avoid technically demanding left SCA revascularisation deep in the thorax.

We aimed to simplify TAR procedures as much as possible for high-risk cases to minimise the risk and complexity of cases by combining TAR with FET following the debranching-first technique. Here, we investigated the efficacy and outcomes of this technique in patients with extensive aortic disease.

## Patients and methods

### Study population

Forty-nine consecutive patients with extensive degenerative aneurysms from the ascending/aortic arch to the descending aorta or chronic aortic dissection receiving left CCA–SCA bypass followed by TAR using a FET in 2016–2021 were enrolled in this study. Acute aortic dissection DeBakey 1 or 2 cases requiring emergency surgery were excluded from this study, as emergency cases did not allow for the waiting interval between the first- and second-stage surgeries. The type of chronic aortic dissection was DeBakey 1 or 3b/3b retrograde (3bR). We used *the Euro*SCORE II as a reference for risk stratification [7]. This was a retrospective analysis to investigate whether the assessed technique can minimise the risk of surgery by simplifying complex TAR procedures for high-risk patients.

### Definition

The disabling stroke was indicated by the modified Rankin scale of > 3 (range 0–6, higher scores indicate severe disability). Cerebral events were defined as transient or permanent, including those patients with new neurological symptoms.

### Procedures

**Fig. 1:**
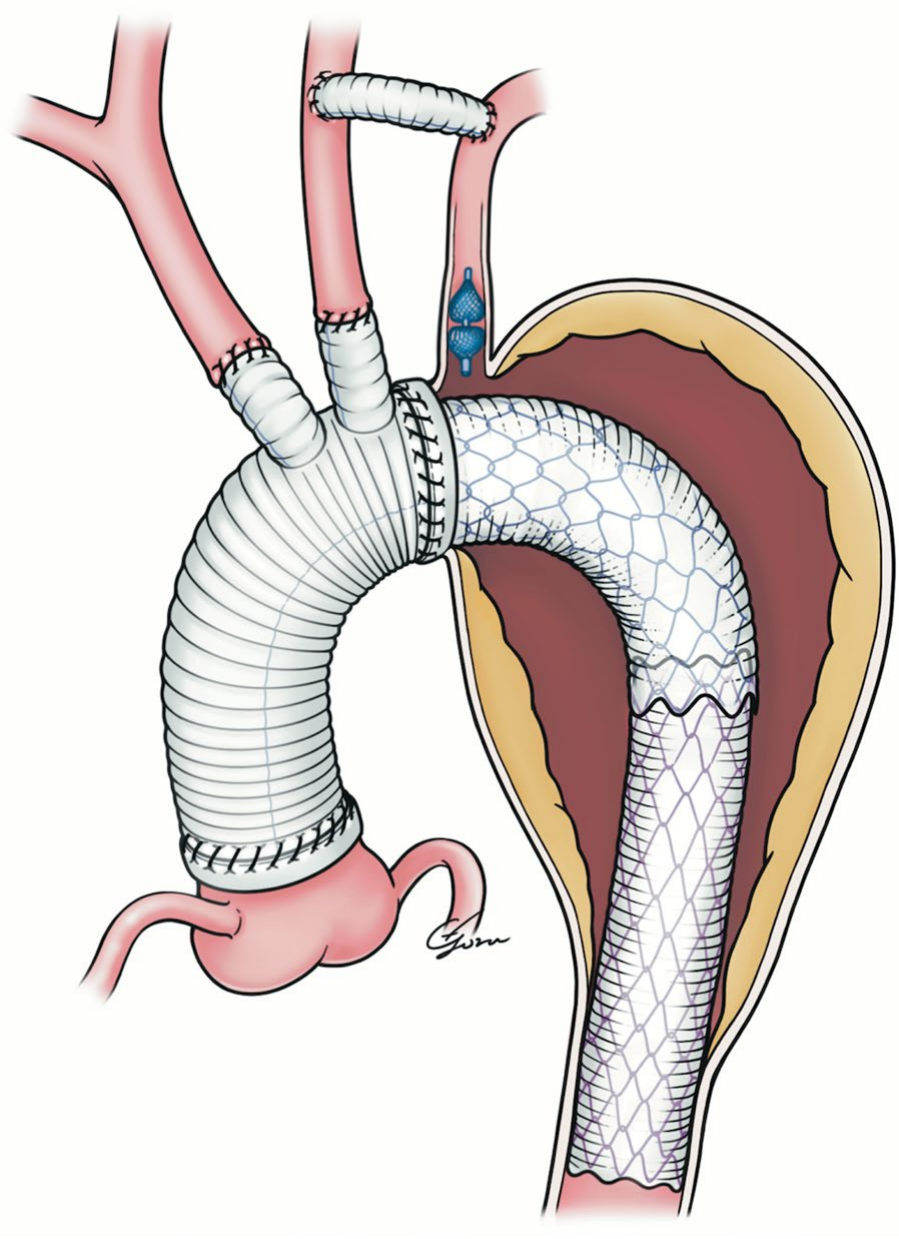
Operative schema of total arch replacement using frozen elephant trunk and neck vessel debranching

First, a left CCA–SCA bypass was performed. A skin incision was made either above or below the clavicle and neck area. A 6- to 8-mm Goretex graft (Propaten® Vascular Graft (W. L. Gore and Associates, Inc., Newark, Delaware, United States)) was tunnelled under the skin and connected to each vessel. A vascular plug (Amplatzer Vascular Plug Abbott Laboratories, Chicago, USA) was placed in the LSA. Angiography was used to confirm graft patency and proper plug placement. Approximately 1 week later, TAR was performed using a FET (Fig. 1). A median sternotomy was performed under general anaesthesia. Arterial lines were placed on both upper extremities to ensure adequate selective cerebral perfusion. Near-infrared spectroscopy was performed to monitor brain circulation. The perfusion route was selected for cannulation, either through the ascending aorta, axillary artery, or femoral artery. Bicaval venous drainage was then performed. Subsequently, the temperature was reduced to 26 °C. Once circulatory arrest was achieved, antegrade selective cerebral perfusion with the brachiocephalic artery and the left CCA was initiated. A combination of antegrade and retrograde cardioplegia was administered to protect the heart. The aortic arch was transected between the left CCA and SCA, through which an open stent graft (J Graft Frozenix®, Japan Lifeline, Tokyo, Japan) was deployed toward the descending aorta. The FET prosthesis, Frozenix®, consists of a Dacron polyester fabric vascular prosthesis with nitinol stents affixed to the inner aspect. The delivery system consisted of a malleable rod that could be advanced into the descending aorta. Preoperative multiplanar reconstruction computed tomography was performed routinely, and the distances between the planned distal anastomosis of the tetrafurcated tube graft for TAR and the target distal end of the FET prosthesis were measured [8]. The size was selected accordingly. For the lumen diameter, 90% of long diameter of the aortic wall, including true and false lumens. For degenerative cases, we chose 110–120% of diameter of the descending aorta.

With the prior left CCA–SCA bypass, the distal anastomosis of the aortic arch was performed at the zone 2 level, connecting the trifurcated aortic arch graft, J-graft® (Japan Lifeline Co., Ltd., Tokyo, Japan) and FET prosthesis which had covered the LSA orifice (Fig. 1). The distal anastomosis of the aortic arch repair was moved close to the heart as opposed to deep in the thorax; thus, it was easier to handle. Following these procedures, additional thoracic endovascular aortic repair (TEVAR) was performed to treat residual descending aortic aneurysms. The proximal landing zone for TEVAR was at the distal end of FET prosthesis, and the distal landing zone for TEVAR was at the proximal descending aorta to cover the residual pathology. As a spinal cord protection and stroke prevention, we kept blood pressure over mean arterial pressure of 80 mmHg when cross-clamping carotid artery while debranching and TEVAR, and also kept hemoglobin level above 10 g/dL during TEVAR.

### End points and follow-up

The primary endpoints were operative mortality, estimated 5-year overall survival, and 5-year aortic event-free survival rates. The secondary endpoints were estimated cumulative incidence rates of planned or unplanned aortic reintervention. The patients were followed up at the outpatient unit of our institute, and an annual computed tomography scan was performed to evaluate aneurysm remodelling. For patients who underwent follow-up at another hospital, contact with primary physicians or telephone interviews were conducted. Loss to follow-up was defined as a lost to follow-up within 30 days after discharge [9]. Accordingly, all patients were followed up.

### Statistical analyses

Follow-up outcomes were estimated using Kaplan–Meier analysis. The 5-year aortic-related mortality, overall cumulative aortic reintervention, and unplanned cumulative aortic reintervention rates were estimated using death as a competing risk. When the sample data were not normally distributed, the variables were expressed as medians and interquartile ranges (IQR). For normally distributed data, variables were expressed as the mean ± standard deviation. We used the IBM SPSS Statistics (version 22.0; IBM Japan, Ltd., Tokyo, Japan) for the statistical analyses.

## Results

Patient characteristics are summarised in Table 1. The patients in this study were predominantly male (81.6%), aged 73.5 ± 8.3 years, with various comorbidities, including diabetes mellitus (28.6%) and hyperlipidaemia (53.1%), and with a history of cerebrovascular events (22.4%) and haemodialysis (8.2%). Aortic pathologies included distal arch degenerative aneurysms (30.6%, 15/49), degenerative aortic arch (36.7%, 18/49), chronic aortic dissection DeBakey 3b or 3bR (24.5%, 12/49), and type 1 (8.1%, 4/49). The average *Euro*SCORE II was 4.7 ± 2.5. The median *Euro*SCORE II was 4.4 [2.8–6.3]. The patency rate of the Left CCA-SCA bypass was 100%, and the rate of stenosis was approximately 15%, with a discrepancy between the right and left upper extremity blood pressure (right > left about 15–20 mmHg). During the follow-up, we did not observe any bypass failure. We observed one case of brachial plexus palsy after debranching. Left arm weakness was noted postoperatively for a month but was cured completely after rehabilitation.

**Table 1:** Baseline characteristics

Preoperative variables	N = 49
Age (year)	73.5 ± 8.3
Female	9 (18.4)
Male	38 (81.6)
Body surface area (m^2^)	1.7 ± 0.2
Hyperlipidemia, n (%)	25 (53.1)
Diabetes mellitus, n (%)	13 (28.6)
Hypertension, n (%)	41 (83.7)
Lung disease, n (%)	4 (8.2)
Peripheral vascular disease, n (%)	9 (18.4)
Arrythmia, n (%)	3 (6.1)
Previous angina, MI, n (%)	6 (12.2)
Previous CVA, n (%)	11 (22.4)
Previous heart surgery, n (%)	5 (10.2)
Haemodialysis, n (%)	4 (8.2)
Serum creatinine (mg/dL)	1.0 [0.7, 1.4]
Ejection fraction (%)	62.8 ± 7.7
Aortic valve pathology, n (%)	15 (30.6)
Mitral valve pathology, n (%)	2 (4.1)
Duration from debranching (days)	9.0 [7.0, 28.0]
*Type of aneurysm*	
Degenerative arch or distal arch	33 (67.3)
Debakey 3b or 3bR	12 (24.5)
Debakey 1	4 (8.2)
EuroSCORE II	4.7 ± 2.5
	4.4 [2.8–6.3]

Values are presented as number (%) or mean (SD) or median [IQR]. MI, myocardial infarction; CVA, cerebrovascular accident. The valve pathology: greater than moderate regurgitation or stenosis was included. Debakey 3bR: aortic dissection Debakey 3b retrograde

Intraoperative data (Table 2) showed a median operation and cardiopulmonary bypass time of 272 [IQR, 250–328] and 203 [IQR, 182–242] min, respectively. Ascending aortic cannulation was performed in 79.2% of patients. However, 10.4% of the patients underwent right SCA cannulation, and 6.3% underwent femoral cannulation. The median length of the open stent graft was 60 mm [IQR, 60–90 mm]. The average lumen diameter was 27 mm. Furthermore, 18.2% of patients required concomitant procedures (coronary artery bypass graft, 4; aortic valve replacement, 3; pulmonary vein isolation, 1). The median duration from the first bypass to TAR was 9 [IQR, 7–28] days. The median lengths of intubation and intensive care unit (ICU) stay were 2.5 [IQR, 1.8–5.0] and 6 [IQR, 4.0–13.5] days, respectively.

**Table 2:** Operative characteristics and postoperative outcomes

*Intraoperative data*		
Operation time (min)	272.0	[250.0, 328.0]
Cannulation site		
Ascending aorta, n (%)	38	(79.2)
Subclavian artery, n (%)	5	(10.4)
Femoral artery, n (%)	3	(6.3)
Open stent graft length (mm)	60.0	[60.0, 90.0]
Concomitant procedures, n (%)	8	(18.2)
Cardiac arrest time (min)	174.0	[154.0, 207.0]
Antegrade cerebral perfusion time (min)	65	[30.1, 74.1]
Circulatory arrest time (min)	25	[12.0, 33.0]
Cardiopulmonary bypass time (min)	203.0	[182.3, 242.5]
Antegrade bilateral carotid perfusion	47	(100.0)
*Postoperative outcome*		
Intubation length (days)	2.5	[1.8, 5.0]
Intensive care unit stay (days)	6.0	[4.0, 13.5]
30-day mortality, n (%)	1	(2.0)
Additional TEVAR, n (%)	15	(30.6)
d-SINE, n (%)	2	(4.1)
*Complications*	18	(36.7)
Stroke	5	(10.2)
Pneumonia	4	(8.1)
Atrial fibrillation	4	(8.1)
Urinary tract infection	2	(4.1)
Renal failure	2	(4.1)
Mediastinal re-exploration	1	(2.0)
Operative mortality	2	(4.1)
5-year all-cause mortality	10	(20.4)

Values are presented as number (%) or median [IQR]. TEVAR, thoracic endovascular aortic repair; d-SINE, distal stent graft-induced new entry

Regarding postoperative outcomes (Table 2), there were two reported operative mortality. The cause of death for the first patient was a rupture of the arch aneurysm measuring > 6 cm after the left CCA–SCA bypass while waiting for second-stage TAR. Another patient developed aspiration pneumonia after TAR and underwent a tracheostomy. However, the patient died of multiple organ failure. The operative mortality rate was 4.1% (2/49). Five patients (10.2%, 5/49) developed stroke. All of them were developed after TAR. The average intubation length was 6.4 days, and the length of ICU stay was 16.2 days for those five patients. All patients were discharged from the hospital. Based on the Rankin scale, one patient had a score of 4+, two had a score of 3+, and two had a score of 2+. The most frequently observed radiological finding was a stroke in the right middle cerebral artery area. The relatively high stroke rate in our cohort led to the investigation of the factors associated with stroke. Logistic regression analysis identified atheroma of the ascending aorta as a risk factor for stroke. We did not observe any case of recurrent nerve injury; although some cases developed swallowing difficulty necessitating speech therapy, it was temporary, and the recurrent nerve was intact. No episodes of paraplegia were observed.

During follow-up, 15 patients (30.6%, 15/49) patients required further TEVAR to treat residual descending aortic aneurysms. No episodes of paraplegia occurred after additional TEVAR. Two (4.1%, 2/49) patients developed distal stent graft-induced new entry (dSINE) due to an open stent graft, requiring intimal tear closure by subsequent TEVAR. An open stent graft has a spring-back force that causes a tear around the distal edge of the open stent, known as dSINE. The mean duration from TAR to additional planned TEVAR was 32.7 days. However, for two patients undergoing TEVAR for dSINE, the duration was 6 months to 2 years, as TEVAR was an unplanned procedure for these two patients. The average level of the thoracic vertebrae at the distal edge of the endoprosthesis (TEVAR) was 8.2. During follow-up, the excluded aneurysm was remodelled or thrombosed, and chronic dissection cases showed a complete thrombosed false lumen in the extent of the aortic area covered by the stent graft down to the descending aorta, but the abdominal aorta was partially thrombosed in most cases. The degenerative aneurysm exhibited a stable sac size or a slight decrease in size.

**Fig. 2:**
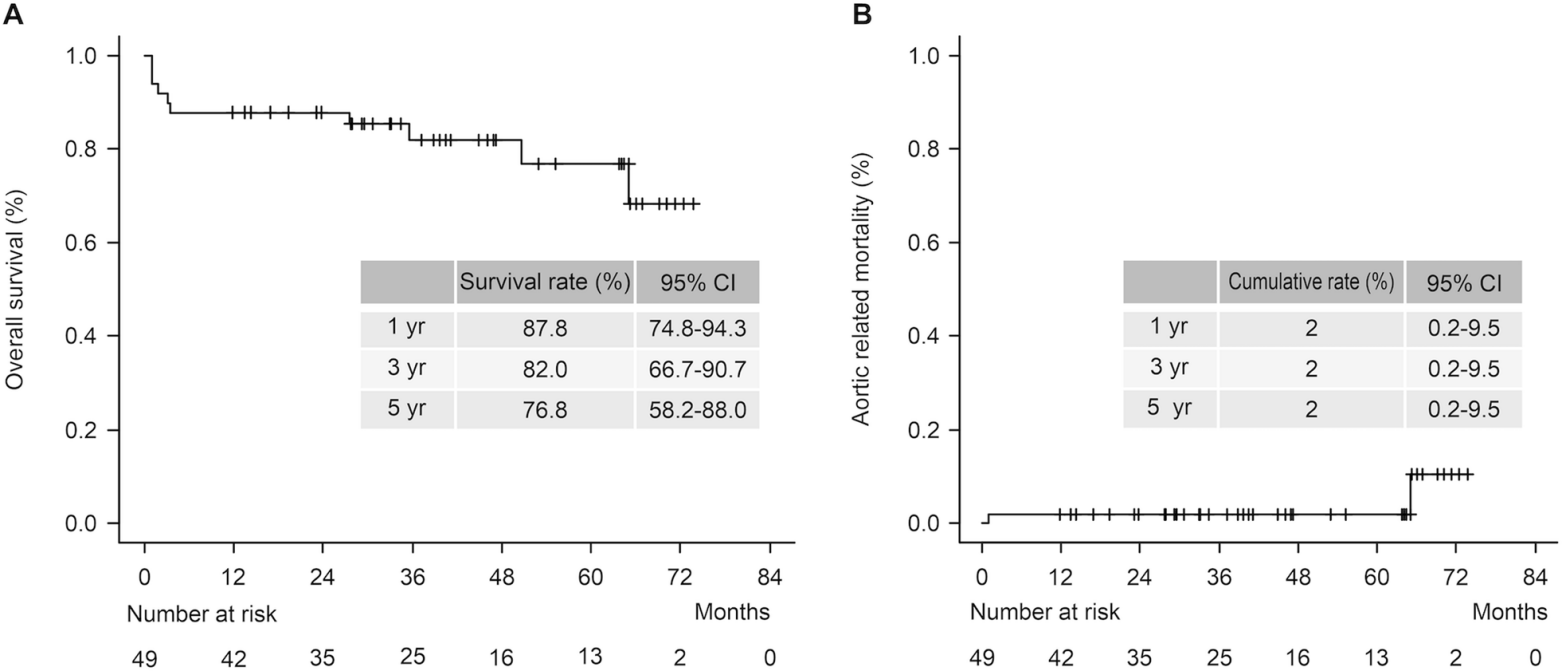
Estimated overall survival (%) (**A**). Estimated cumulative aortic-related mortality (%) (**B**)

**Fig. 3:**
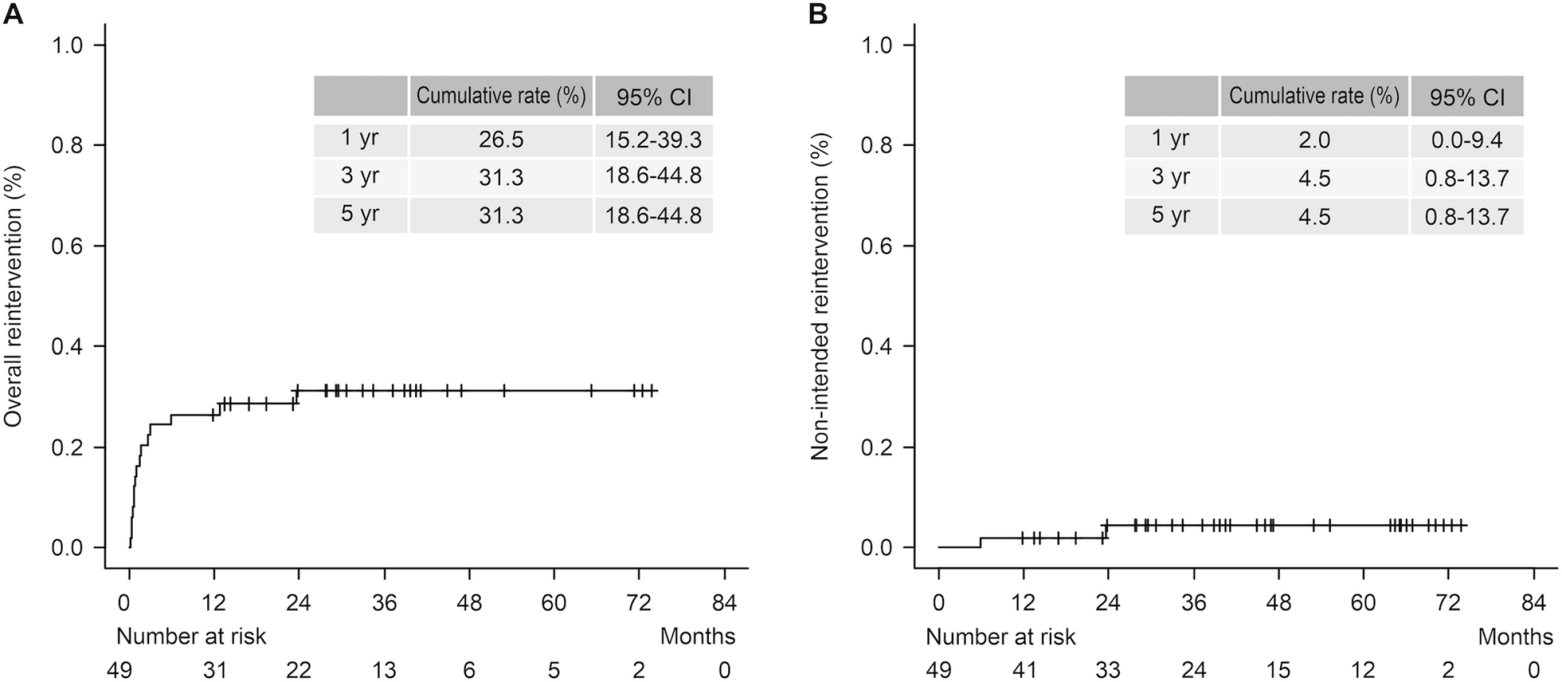
Estimated overall cumulative incidence of reintervention (%) (**A**). Unplanned reintervention (%) (**B**)

Regarding follow-up outcomes, the estimated 5-year overall survival was 76.8% (Fig. 2A). Causes of death were pneumonia (n = 2), renal failure (n = 1), arrhythmia (n = 1), sepsis (n = 2), thoracic aneurysm rupture (n = 1), pseudoaneurysm rupture 1, lung = 1). The estimated 5-year aortic-related mortality rate was 2% (Fig. 2B). Accordingly, the estimated 5-year aortic event-free survival rate is 98%. The average observation period for overall survival and aortic-related mortality was 38.4 ± 22.5 months. The estimated 5-year overall cumulative aortic reintervention rate was 31.3% (Fig. 3A). The estimated cumulative rate of non-intended reintervention was 4.5% at 5 years (Fig. 3B). The average observation period for the overall reintervention rate was 23.8 ± 22.0 months.

## Discussion

In this study, we investigated the postoperative outcomes of the debranching-first technique (left CCA–SCA bypass) followed by TAR using FET as a two-stage procedure for extensive aortic pathology: degenerative aneurysms extending from the aortic arch to the descending aorta or DeBakey type 1 or 3b/3bR chronic aortic dissection. We achieved reasonable 5-year mortality and morbidity rates without any paraplegic events in high-risk patients, although a relatively high rate of stroke events was noted. Multivariate analysis revealed that atheroma in the ascending aorta contributed significantly to stroke events. All stroke events occurred after TAR. Manipulation of the ascending aorta during TAR is thought to cause stroke. One of the reasons we performed TAR following a debranching procedure other than TEVAR following debranching of the neck vessel was the quality of the aortic wall.

TAR using a FET for the closure of primary entry at the distal arch due to aortic type A dissection received a class IIa recommendation from the Vascular Domain of the European Association for Cardio-Thoracic Surgery in 2015 [10]. We further modified the surgical strategy by combining TAR using FET with the debranching-first technique and possible subsequent TEVAR.

### Debranching-first technique (left common carotid artery–subclavian artery bypass)

Promising preliminary results of debranching supra-aortic vessels followed by TAR with modified FET endoprosthesis have been reported for the treatment of aortic arch pathology [6, 11, 12]. The following are several advantages of the debranching-first technique.

- There was continuous blood flow to both hemispheres through a debranching bypass during the entire procedure without any flow interruption. The left CCA–SCA bypass enables continuous blood supply to the anterior spinal artery through the left thoracic and vertebral artery collaterals, which increases spinal cord protection under hypothermic circulatory arrest [13]. Furthermore, direct cannulation of neck vessels for selective cerebral perfusion can be minimised, thereby decreasing the risk of embolisation [6, 13].
- Technically demanding left SCA reconstruction deep into the thorax can be avoided. Left SCA anastomosis is technically demanding due to its deep thoracic position [14]. Separating left SCA revascularisation from aortic arch repair (staged procedures) reduces the scope of operation [15].
- The debranching-first technique extends the landing zone for endoprosthesis, facilitating more proximal FET placement, which allows zone 2 distal anastomosis of aortic arch repair.
- The combination of proximalised FET and avoidance of left SCA reconstruction in the deep thorax reduces the rate of recurrent nerve injury and circulatory arrest time, leading to a lower stroke rate [16].
- A significant increase in the 5-year survival using the FET proximalisation concept (zone ≤ 2) (72.9%) in comparison to the zone 3 arch repair technique (59.5%) was reported (HR 0.7, 95% CI 0.4–1.0, *P* = 0.036) [17].

### Total arch replacement using a frozen elephant trunk following debranching-first technique ± subsequent TEVAR

In general, FET helps avoid aneurysmal cavities, entry tears, and reroute true lumen flow by alleviating compression from the false lumen and promoting thrombosis of the false lumen, which ensues until the level of prosthesis placement in most cases [10, 18, 19]. However, adverse events such as paraplegia and dSINE are disadvantages of FET [20]. Our study had no paraplegia events, and only 4.1% (2/49) of dSINE cases were observed after TAR. Plausible reasons for the absence of paraplegia events are as follows:

- Short-length open stents
  We intentionally used relatively short-length open stents (median length, 6 cm) to minimise paraplegia and were willing to perform additional TEVAR instead as a third-stage intervention to cover the residual aortopathy (26.5%, 13/49). In our study, the average level of the thoracic vertebrae at the distal edge of the endoprosthesis (TEVAR) was 8.2. FET placement below thoracic vertebrae level 10 is a known risk factor for spinal cord injury (SCI) [21, 22].
- Preserving continuous vertebral artery collateral network toward the anterior spinal artery throughout the TAR procedure due to Left CCA-SCA bypass [13].
- Staged surgeries allow collateral network development for spinal cord perfusion.
  In the case of TEVAR for thoracoabdominal aneurysms, the so-called “paraplegia prevention branch” was attached to the endoprosthesis [23]. As a result, the temporary endoleak persisted, which allowed perfusion of the intercostal artery toward the spinal cord through endoleak and 3–4 weeks later, the endoleak was closed. This interval has been reported to be helpful in the development of collateral circulation, working as a SCI prevention measure.
- The second TEVAR, covering vulnerable areas for SCI, can be performed in haemodynamically optimal conditions

If FET is deployed to the distal arch during a circulatory arrest while performing TAR, hypotension status would potentially cause SCI as hypotension during the procedure and the perioperative period is a risk factor for SCI [24]. The cause of paraplegia is multifactorial and all the bullet points we mentioned above are related to paraplegia. The third aortic intervention (planned TEVAR) was performed in a relatively short period (average duration, 32.7 days). Therefore, we could overcome the disadvantages of staged surgery by minimising aortic events between the second and third interventions. The estimated 5-year survival rate was 76.8%, and the 5-year cumulative aortic-related mortality was only 2% (Fig. 2). The survival curve showed a gradual decline, suggesting that 30-day mortality due to the main surgery was considerably low. The estimated 5-year overall versus non-intended cumulative rate for reintervention was 31.3 versus 4.3%, implying that additional planned TEVAR may have saved patients effectively (Fig. 3). Our multiple stages of open aortic surgery with endovascular intervention may be effective in achieving the goal of improving the outcome in high-risk patients, which is consistent with a previous study using a collared elephant trunk prosthesis for TAR and subsequent TEVARs for diffuse thoracic aneurysm [25]. TEVAR had been intentionally delayed after TAR, which was followed by additional TEVAR to cover the residual lesion in the aorta (so-called three steps of delayed frozen elephant trunk approach). With this delay, TEVAR can be performed in good haemodynamic condition with a reasonable collateral network for spinal cord perfusion.

Other researchers have also reported favourable results after supra-aortic debranching in combination with FET [6, 16]. The extended variety of new FET stent grafts and the debranching techniques facilitating anastomosis proximalisation up to zone 0 may allow for a more patient-tailored approach and improve the results, especially in multimorbid patients [17].

### Limitations

The present study was a retrospective cohort study of surgical interventions with a small number of patients. There was no control group with a fair number of patients to compare standard TAR versus TAR plus FET following debranching of the neck vessels. Further large-scale studies with long-term follow-up and propensity-matched analyses are warranted to confirm these findings. Regarding the two-stage operation, a certain number of patients were lost to follow-up or died while waiting for the second-stage surgery [2, 3]. Therefore, it may be rational to perform the debranching procedure and TAR simultaneously. However, this could lead to longer operation times and higher comorbidity rates. Considering the high-risk cohort, staging interventions can minimise the operation risk. Moreover, we performed two operations during similar hospitalisations without discharging the patients, which can reduce the risk of loss to follow-up and interval morbidity. Regarding the risk assessment of postoperative patients, we included patients with a relatively high incidence of peripheral vascular disease (18.4%), previous cerebrovascular accidents (22.4%), and haemodialysis (8.2%). Although the average *Euro*SCORE II score was 4.7 ± 2.5 in our study and a score greater than 6 was considered high-risk, it would be reasonable to say that these are high-risk patients. Moreover, *Euro*SCORE II may not be appropriate for estimating postoperative risk; therefore, we used it as an adjunctive tool along with other preoperative variables.

## Conclusion

The combination of the debranching-first technique followed by TAR using a FET and possible subsequent TEVAR represents a less technically demanding surgery for diffuse thoracic aneurysm. Our data support this statement and suggest that this combination may be useful for managing high-risk patients, as this technique mitigates the operative risks by staging open and endovascular intervention alternatively, which sets the stage for an optimal environment for subsequent endovascular intervention, leading to lower incidence rates of SCI with minimum aortic events.

## Data Availability

The data analysed in this article may be shared upon request sent to the corresponding author.

## Abbreviations

TAR: Total arch replacement
FET: Frozen elephant trunk
SCA: Subclavian artery
LSA: Left subclavian artery
CCA: Common carotid artery
TEVAR: Thoracic endovascular aortic repair
dSINE: Distal stent graft-induced new entry
SCI: Spinal cord injury
